# Supplementary material for: Chronic exposure to odors at naturally occurring concentrations triggers limited plasticity in early stages of Drosophila olfactory processing
Source: eLife. 2023 May 30;12:e85443. doi: 10.7554/eLife.85443 (PMC10229125; doi:10.7554/eLife.85443)
Supplement: Supplementary file 1. — Where only a subset of stimuli is plotted due to space constraints, bolded entries correspond to the stimuli represented in the figure. Solvent is paraffin oil. [file elife-85443-supp1.docx]

**SUPPLEMENTARY FILE 1**

**Supplemental Table 1: Complete genotypes and *n* for all experiments.**

Where only a subset of stimuli is plotted due to space constraints, bolded entries correspond to the stimuli represented in the figure. Solvent is paraffin oil.

| FIGURE | GeNOTYPE | EXPERIMENTAL  GROUP | STIMULUS | No. FLIES (*N*) |
| --- | --- | --- | --- | --- |
| 1C-D | *NP3481-Gal4, UAS-CD8:GFP* (X) | E2-hexenal | solvent  E2-hexenal, 10^-7^ | 1  1 |
| 2B-F  2Q | *NP3481-Gal4, UAS-CD8:GFP* (X) | E2-hexenal exposed | solvent  E2-hexenal, 10^-12^  E2-hexenal, 10^-11^  **E2-hexenal, 10^-10^**  **E2-hexenal, 10^-9^**  **E2-hexenal, 10^-8^**  **E2-hexenal, 10^-7^**  E2-hexenal, 10^-6^  E2-hexenal, 10^-5^ | 3  3  5  **9**  **10**  **11**  **12**  10  6 |
|  |  | solvent exposed | solvent  E2-hexenal, 10^-12^  E2-hexenal, 10^-11^  **E2-hexenal, 10^-10^**  **E2-hexenal, 10^-9^**  **E2-hexenal, 10^-8^**  **E2-hexenal, 10^-7^**  E2-hexenal, 10^-6^  E2-hexenal, 10^-5^ | 3  3  3  **8**  **7**  **19**  **19**  14  9 |
| 2H-J  2R  3F | *NP3481-Gal4, UAS-CD8:GFP* (X) | 2-butanone exposed | 2-butanone, 10^-8^  **2-butanone, 10^-7^**  **2-butanone, 10^-6^**  **2-butanone, 10^-5^**  **2-butanone, 10^-4^** | 4  **11**  **12**  **13**  **12** |
|  |  | solvent exposed | 2-butanone, 10^-8^  **2-butanone, 10^-7^**  **2-butanone, 10^-6^**  **2-butanone, 10^-5^**  **2-butanone, 10^-4^** | 6  **16**  **13**  **14**  **11** |
| 2L-P  2S | *UAS-CD8:GFP* (X)*; MZ612-Gal4, UAS-CD8:GFP* (II) | geranyl acetate exposed | geranyl acetate, 10^-7^  geranyl acetate, 10^-6^  geranyl acetate, 10^-5^  geranyl acetate, 10^-4^ | 7  9  9  8 |
|  |  | solvent exposed | geranyl acetate, 10^-7^  geranyl acetate, 10^-6^  geranyl acetate, 10^-5^  geranyl acetate, 10^-4^ | 10  11  11  8 |
| 3B-E  3G-H | *NP3481-Gal4, UAS-CD8:GFP* (X) | E2-hexenal exposed | 2-butanone, 10^-7^  2-butanone, 10^-6^  2-butanone, 10^-5^  2-butanone, 10^-4^ | 6  8  7  5 |
|  |  | solvent exposed | 2-butanone, 10^-7^  2-butanone, 10^-6^  2-butanone, 10^-5^  2-butanone, 10^-4^ | 16  13  14  11 |
| 3J-N | *UAS-CD8:GFP* (X)*; MZ612-Gal4, UAS-CD8:GFP* (II) | E2-hexenal exposed | geranyl acetate, 10^-7^  geranyl acetate, 10^-6^  geranyl acetate, 10^-5^  geranyl acetate, 10^-4^ | 5  5  5  5 |
|  |  | solvent exposed | geranyl acetate, 10^-7^  geranyl acetate, 10^-6^  geranyl acetate, 10^-5^  geranyl acetate, 10^-4^ | 10  11  11  8 |
| 4B-F | *UAS-CD8:GFP* (X)*; MZ612-Gal4, UAS-CD8:GFP* (II) | 20% geranyl acetate exposed | geranyl acetate, 10^-7^  geranyl acetate, 10^-6^  geranyl acetate, 10^-5^  geranyl acetate, 10^-4^ | 5  5  5  6 |
|  |  | solvent exposed | geranyl acetate, 10^-7^  geranyl acetate, 10^-6^  geranyl acetate, 10^-5^  geranyl acetate, 10^-4^ | 5  5  5  6 |
| 4H-L | *NP3481-Gal4, UAS-CD8:GFP* (X) | 20% geranyl acetate exposed | valeric acid, 5x10^-4^  valeric acid, 10^-3^  valeric acid, 5x10^-3^  valeric acid, 10^-2^ | 6  6  6  6 |
|  |  | solvent exposed | valeric acid, 5x10^-4^  valeric acid, 10^-3^  valeric acid, 5x10^-3^  valeric acid, 10^-2^ | 6  6  6  5 |
| 5B-C | *NP3481-Gal4, UAS-CD8:GFP* (X) | 2-butanone exposed | *pentyl acetate, 10^-3^* +2-butanone, 10^-8^  +2-butanone, 10^-7^  +2-butanone, 10^-6^  +2-butanone, 10^-5^  +2-butanone, 10^-4^ | 3  7  6  7  6 |
|  |  | solvent exposed | *pentyl acetate, 10^-3^* +2-butanone, 10^-8^  +2-butanone, 10^-7^  +2-butanone, 10^-6^  +2-butanone, 10^-5^  +2-butanone, 10^-4^ | 6  6  6  7  5 |
| 5E-F | *NP3481-Gal4, UAS-CD8:GFP* (X) | E2-hexenal exposed | *pentyl acetate, 10^-3^* +2-butanone, 10^-8^  +2-butanone, 10^-7^  +2-butanone, 10^-6^  +2-butanone, 10^-5^  +2-butanone, 10^-4^ | 3  3  4  3  3 |
|  |  | solvent exposed | *pentyl acetate, 10^-3^* +2-butanone, 10^-8^  +2-butanone, 10^-7^  +2-butanone, 10^-6^  +2-butanone, 10^-5^  +2-butanone, 10^-4^ | 6  6  6  7  5 |
| S5A | *+/UAS-brp.S-mStraw* (II)*; 20XUAS-CD8:GFP/NP3056-Gal4* (III) | solvent exposed | NA | 1 |
| S5B  S5D  S5F  S5H | *+/UAS-brp.S-mStraw* (II)*; 20XUAS-CD8:GFP/NP3056-Gal4* (III) | 2-butanone exposed | NA | 13 |
|  |  | solvent exposed | NA | 10 |
| S5C  S5E  S5G  S5I | *+/UAS-brp.S-mStraw* (II)*; 20XUAS-CD8:GFP/NP3056-Gal4* (III) | E2-hexenal exposed | NA | 11 |
|  |  | solvent exposed | NA | 8 |
| 6B  6C | *NP3481-Gal4, UAS-CD8:GFP* (X) | E2-hexenal exposed | solvent  E2-hexenal, 10^-12^  E2-hexenal, 10^-11^  **E2-hexenal, 10^-10^**  **E2-hexenal, 10^-9^**  **E2-hexenal, 10^-8^**  **E2-hexenal, 10^-7^**  E2-hexenal, 10^-6^  E2-hexenal, 10^-5^  E2-hexenal, 10^-4^ | 12  11  11  10  10  8  9  9  9  10 |
|  |  | solvent exposed | solvent  E2-hexenal, 10^-12^  E2-hexenal, 10^-11^  **E2-hexenal, 10^-10^**  **E2-hexenal, 10^-9^**  **E2-hexenal, 10^-8^**  **E2-hexenal, 10^-7^**  E2-hexenal, 10^-6^  E2-hexenal, 10^-5^  E2-hexenal, 10^-4^ | 9  9  9  8  8  9  9  7  6  6 |
| 6E  6F | *NP3481-Gal4, UAS-CD8:GFP* (X) | 2-butanone exposed | solvent  2-butanone, 10^-10^  2-butanone, 10^-9^  2-butanone, 10^-8^  **2-butanone, 10^-7^**  **2-butanone, 10^-6^**  **2-butanone, 10^-5^**  **2-butanone, 10^-4^**  2-butanone, 10^-3^ | 6  9  9  9  10  10  9  10  9 |
|  |  | solvent exposed | solvent  2-butanone, 10^-10^  2-butanone, 10^-9^  2-butanone, 10^-8^  **2-butanone, 10^-7^**  **2-butanone, 10^-6^**  **2-butanone, 10^-5^**  **2-butanone, 10^-4^**  2-butanone, 10^-3^ | 9  11  10  10  10  9  9  9  9 |
| 6H  6I | *NP3481-Gal4, UAS-CD8:GFP* (X) | E2-hexenal exposed | solvent  2-butanone, 10^-10^  2-butanone, 10^-9^  2-butanone, 10^-8^  **2-butanone, 10^-7^**  **2-butanone, 10^-6^**  **2-butanone, 10^-5^**  **2-butanone, 10^-4^**  2-butanone, 10^-3^ | 5  9  9  9  9  9  8  8  9 |
|  |  | solvent exposed | solvent  2-butanone, 10^-10^  2-butanone, 10^-9^  2-butanone, 10^-8^  **2-butanone, 10^-7^**  **2-butanone, 10^-6^**  **2-butanone, 10^-5^**  **2-butanone, 10^-4^**  2-butanone, 10^-3^ | 9  11  10  10  10  9  9  9  9 |
| 6K | *+/Or7a-Gal4*(KI) (X)*;* +/*20XUAS-IVS-syn21-opGCaMP6f-p10* (II) | solvent exposed | E2-hexenal, 10^-7^ | 1 |
| 6L  6M | *+/Or7a-Gal4*(KI) (X)*;* +/*20XUAS-IVS-syn21-opGCaMP6f-p10* (II) | E2-hexenal exposed | solvent  E2-hexenal, 10^-12^  E2-hexenal, 10^-11^  **E2-hexenal, 10^-10^**  **E2-hexenal, 10^-9^**  **E2-hexenal, 10^-8^**  **E2-hexenal, 10^-7^**  E2-hexenal, 10^-6^ | 8  6  8  8  8  8  7  8 |
|  |  | solvent exposed | solvent  E2-hexenal, 10^-12^  E2-hexenal, 10^-11^  **E2-hexenal, 10^-10^**  **E2-hexenal, 10^-9^**  **E2-hexenal, 10^-8^**  **E2-hexenal, 10^-7^**  E2-hexenal, 10^-6^ | 8  6  8  8  8  8  7  8 |
| 7B-C | *NP3481-Gal4, UAS-CD8:GFP* (X) | E2-hexenal exposed | NA | 4 |
|  |  | solvent exposed | NA | 4 |
| 7E | *NP3481-Gal4, UAS-CD8:GFP* (X)*;* +/*13xlexAop2-IVS-CsChrimson.mVenus* (II); +/*Or7a-lexA* (III) | solvent exposed | NA | 1 |
| 7F-G | *NP3481-Gal4, UAS-CD8:GFP* (X)*;* +/*13xlexAop2-IVS-CsChrimson.mVenus* (II); +/*Or7a-lexA* (III) | E2-hexenal exposed | NA | 5 |
|  |  | solvent exposed | NA | 7 |
| 7I-J | *UAS-CD8:GFP* (X)*; MZ612-Gal4, UAS-CD8:GFP* (II) | E2-hexenal exposed | solvent  pentyl acetate  2-heptanone  E2-hexenal  isobutyl acetate  *p*-cresol  2-butanone | 3  3  3  3  3  3  3 |
|  |  | solvent exposed | solvent  pentyl acetate  2-heptanone  E2-hexenal  isobutyl acetate  *p*-cresol  2-butanone | 3  5  4  4  4  4  3 |
